# Classification of Trauma-Related Outcomes in US Veterans Using Magnetoencephalography (MEG)

**DOI:** 10.29245/2572.942x/2021/1.1279

**Published:** 2021

**Authors:** Lisa M. James, Brian E. Enghdal, Arthur C. Leuthold, Apostolos P. Georgopoulos

**Affiliations:** 1 The PTSD Research Group, Brain Sciences Center, Department of Veterans Affairs Health Care System, Minneapolis, MN, USA; 2 Department of Neuroscience, University of Minnesota Medical School, Minneapolis, MN, USA; 3 Department of Psychiatry, University of Minnesota Medical School, Minneapolis, MN, USA; 4 Center for Cognitive Sciences, University of Minnesota, Minneapolis, MN, USA; 5 Department of Psychology, University of Minnesota, Minneapolis, MN, USA; 6 Department of Neurology, University of Minnesota Medical School, Minneapolis, MN, USA

**Keywords:** Posttraumatic Stress Disorder, Subthreshold Posttraumatic Stress Disorder, Recovery, Magnetoencephalography, Classification, Biomarker

## Abstract

Previous research has demonstrated highly accurate classification of veterans with posttraumatic stress disorder (PTSD) and controls based on synchronous neural interactions (SNI), highlighting the utility of SNI as a biomarker of PTSD. Here we extend that research to classify additional trauma-related outcomes including subthreshold PTSD, partial recovery, and full recovery according to SNI. A total of 219 U.S. veterans completed diagnostic interviews and underwent a magnetoencephalography (MEG) scan from which SNI was computed. Linear discriminant analysis was used to classify the PTSD and control brains, achieving 100% accuracy. That discriminant function was then used to classify each brain in the subthreshold PTSD, partial recovery, and full recovery diagnostic groups as PTSD or Control. All of the subthreshold PTSD diagnostic group were classified as PTSD, as were three-quarters of the partial recovery group. Findings regarding the full recovery group were mixed, documenting variability in the functional brain status of PTSD recovery. The results of the present study add to the literature supporting the discriminatory power of MEG SNI and demonstrate the utility of SNI as a biomarker of various PTSD-related trajectories.

## Introduction

Service members are at elevated risk for posttraumatic stress disorder (PTSD), a psychiatric condition that some people experience as a result of exposure to potentially traumatic experiences. PTSD symptoms include intrusive recollections or re-experiencing of the traumatic event, avoidance of trauma reminders, emotional numbing, and hyperarousal^1^. These symptoms, in addition to co-occurring physical and mental health problems, can result in increased health care use and significant impairment in social and occupational functioning^2–5^. The prevalence of PTSD varies widely due to sample and methodological differences with estimates suggesting that 10–30% of US veterans meet lifetime criteria for PTSD^6–9^. While a significant number of service members are affected by PTSD, these figures suggest that the vast majority experience alternative outcomes following exposure to potentially traumatic events.

One common outcome following exposure to potentially traumatic events is subthreshold PTSD. In order to receive a diagnosis of PTSD, an individual must endorse a specific number of symptoms in each of several symptom domains^1^. Thus, an individual may report numerous PTSD symptoms and exhibit significant trauma-related distress, and yet not meet criteria for a PTSD diagnosis if that person does not report enough symptoms in all of the required domains. The absence of PTSD diagnosis, however, does not equate with lack of serious sequelae. Indeed, subthreshold PTSD is associated with significant clinical impairment^10^ and high rates of co-occurring mental and physical health conditions^11,12^. Furthermore, subthreshold PTSD has been found to be as common or even more common than full-criteria PTSD^13^. Consequently, subthreshold PTSD is increasingly recognized as an important research and clinical focus.

Sustained impairment associated with PTSD or subthreshold PTSD represents one outcome following exposure to potentially traumatic event; however, decades of research on adjustment following exposure to potentially traumatic events have identified several distinct trajectories, the most common of which are resilience and recovery^14–16^. Resilience, which reflects stable psychological and physical health before and after potential trauma exposure, is the modal outcome following trauma exposure, even among military personnel^17,18^. Recovery, the second most common outcome following trauma exposure, reflects initial distress or impairment that may or may not meet diagnostic threshold followed by return to baseline over time. This is reflected in the relatively higher rates of lifetime vs current PTSD rates^5^. Thus, there is considerable individual variability in trauma-related trajectories ranging from minimal impacts to chronic, debilitating effects.

Investigation of trauma outcomes, however, is hampered by reliance on self-report of symptoms. For service members in particular, several factors may influence under- or over-reporting of symptoms including factors related to military culture and contextual factors (e.g., career impacts), help-seeking incentives (e.g., compensation) or deterrents (e.g., stigma), and features of the trauma-response itself (e.g., avoidance, shame, blame)^19^. In addition, individuals may misattribute symptoms of other disorders such as depression or alcohol use disorders to PTSD^19^. In light of the many factors that may influence symptom reporting, identification of objective indicators of PTSD is especially appealing in order to improve diagnostic accuracy and enhance treatment planning and delivery^20^.

Several PTSD biomarkers with varying degrees of validity and replicability have been identified^21^. However, not only should a biomarker be highly accurate and replicable but also be useful for tracking treatment response^20^. One promising PTSD biomarker is magnetoencephalography (MEG)-based synchronous neural interactions (SNI^22^). Previous research in our lab has demonstrated that SNIs distinguish individuals with PTSD from healthy controls with >90% accuracy^23^, and provide highly accurate classification of PTSD even in individuals with comorbid psychiatric diagnoses^24^. In particular, neural interactions involving right superior temporal and occipital-parietal regions characterize cortical miscommunication associated with PTSD^25^. Furthermore, the same pattern, although attenuated, was observed for those with PTSD in remission, suggesting the potential presence of a neural “scar” associated with current or historical PTSD. Subsequent MEG research in other laboratories has further demonstrated accurate classification of PTSD based on neural synchrony^26^, providing additional evidence regarding the utility of MEG-based tests for classification. Here we aim to extend this line of research by moving beyond classification of PTSD vs controls to using MEG SNI to classify other trauma-related outcomes including subthreshold PTSD, partial recovery, and full recovery.

## Materials and Methods

### Study participants

A total of 219 veterans (29 women) participated in this study as paid volunteers. The study protocol was approved by the Minneapolis VAHCS institutional review board and informed consent was obtained prior to the study. Exclusionary criteria included cardiac pacemakers or implanted ferrous metal, central nervous system disorders (e.g. Parkinson’s disease, cerebrovascular accidents, etc.), chronic pain, psychosis, bipolar disorder, current alcohol or drug dependence, or traumatic brain injury. Recruitment targeted healthy control veterans and veterans with current or history of PTSD in the absence of other Axis I diagnoses. All participants were evaluated by doctoral level psychologists according to DSM-IV-TR diagnostic criteria^27^. Current and lifetime PTSD was assessed with the Clinician Administered PTSD Scale (CAPS^28^) or the Structured Clinical Interview for DSM-IV-TR Axis I disorders^29^. In light of research demonstrating wide-ranging reactions to trauma, emotional responses other than intense fear, helplessness, or horror were accepted for criterion A2^30^. PTSD status was determined using the SCID Symptom Calibration method (SXCAL^31^) which provides empirical cut points that permit conversion of continuous CAPS symptom scores to dichotomous scores. The SXCAL method minimizes false-positive and false-negatives and is the preferred CAPS scoring method when differential diagnosis is the goal^31^. Other Axis I disorders were evaluated using the Structured Clinical Interview for DSM-IV-TR^29^. None of the participants included here met criteria for Axis I disorders except for PTSD. Five PTSD-related groups were distinguished based on the diagnostic interview, as follows. (a) The control group (N = 87; 7 women) comprised veterans who did not meet current or historic criteria for any mental health condition; (b) the PTSD group (N = 88; 14 women) met current criteria for PTSD; (c) the subthreshold PTSD group (N = 13; 3 women) included veterans who reported current symptoms of PTSD that did not meet the full diagnostic criteria for PTSD currently or historically; (d) the partially recovered PTSD group (N = 19; 1 woman) included veterans who had met full criteria for PTSD historically and continue to report some symptoms of PTSD but did not meet full criteria for PTSD at the time of assessment; and (e) the fully recovered PTSD group (N = 12; 4 women) included veterans who had historically met full criteria for PTSD and no longer report symptoms consistent with PTSD. The mean (± SEM) age was 55.0 ± 1.70 y for the control group (N = 87), 51.6 ± 1.53 y for the PTSD group (N = 88), 49.8 ± 4.28 y for the subthreshold PTSD group (N = 13), 57.1 ± 3.12 y for the partially recovered PTSD group (N = 19), and 40.4 ± 4.49 y for the fully recovered PTSD group (N = 12).

Participants also completed the Posttraumatic Stress Disorder Checklist for DSM-5 (PCL-5)^32^, a 20-item self-report measure that assesses the presence and severity of PTSD symptoms in accordance with the DSM-5 criteria for PTSD. PCL-5 scores were used to evaluate the relation between PTSD severity and a distance classification measure (see below).

### MEG data acquisition

All participants underwent a MEG scan. As described previously^22^, subjects lay supine within the electromagnetically shielded chamber and fixated their eyes on a spot 65 cm in front of them, for 60s. MEG data were acquired using a 248-channel axial gradiometer system (Magnes 3600WH, 4-D Neuroimaging, San Diego, CA), band-filtered between 0.1 and 400 Hz, and sampled at 1017.25 Hz. Data with artifacts (e.g. from excessive subject motion) were eliminated from further analysis.

### Data analysis

Standard statistical methods were used to analyze the data, including analysis of variance (ANOVA), linear regression, Pearson correlation, and linear discriminant analysis (LDA). The following packages were employed: IBM-SPSS statistical package, version 23^33^, Matlab (version R2015b^34^), and ad hoc Fortran computer programs employing the International Mathematics and Statistics Library (IMSL; Rogue Wave Software, Louisville, CO, USA) statistical and mathematical libraries.

### MEG data processing

Processing of the raw MEG series was performed using programs in Python^35^. Single trial MEG time series from all sensors underwent ‘prewhitening’^36^ using a (50,1,3) ARIMA model to obtain innovations (i.e. residuals)^35^. All possible pairwise zero-lag crosscorrelations (N = 30,628, given 248 sensors) were computed between the prewhitened MEG time series. Finally, the partial zero-lag crosscorrelations $PCC_{ij}^{0}$ (SNI) between i and j sensors were computed for all sensor pairs. $PCC_{ij}^{0}$ was transformed to $z_{ij}^{0}$ using Fisher’s (36) z-transformation to normalize its distribution:

(1) $$\text{SNI}=z_{ij}^{0}=\text{atanh}\left(PCC_{ij}^{0}\right)$$

### LDA

In this analysis, we used the functional brain patterns (SNI^22^) to assess the status of subthreshold, partially recovered and fully recovered PTSD participants and assign them to the Control or PTSD group. For that purpose, we used the age- and gender-adjusted SNIs in a linear discriminant analysis, as follows. For each brain, there were 247 SNIs available for each one of the 248 sensors. For each sensor, we used the maximum and minimum SNI value^38^ as input (N = 248 × 2 = 496 predictors) to a stepwise LDA to classify control and PTSD brains. This analysis yielded 100% correct classification of control and PTSD brains (see below). Hence, we used that discriminant function to classify each brain in the three remaining groups (subthreshold, partially recovered, and fully recovered PTSD). For each case (brain), we retained the discriminant score, the probability of classification to a group and the ${\text{D}}^{2}$ Mahalanobis distances of each case from the center of the control and PTSD group centroid clusters. Finally, we normalized the ${\text{D}}^{2}$ values to be in the 0–1 range:

(2) $$D_{{control}^{\prime }}^{2}=\frac{D_{control}^{2}}{D_{control}^{2}+D_{PTSD}^{2}}$$

(3) $$D_{{PTSD}^{\prime }}^{2}=\frac{D_{PTSD}^{2}}{D_{control}^{2}+D_{PTSD}^{2}}$$

## Results

### Classification of control and PTSD brains

**Figure 1.**
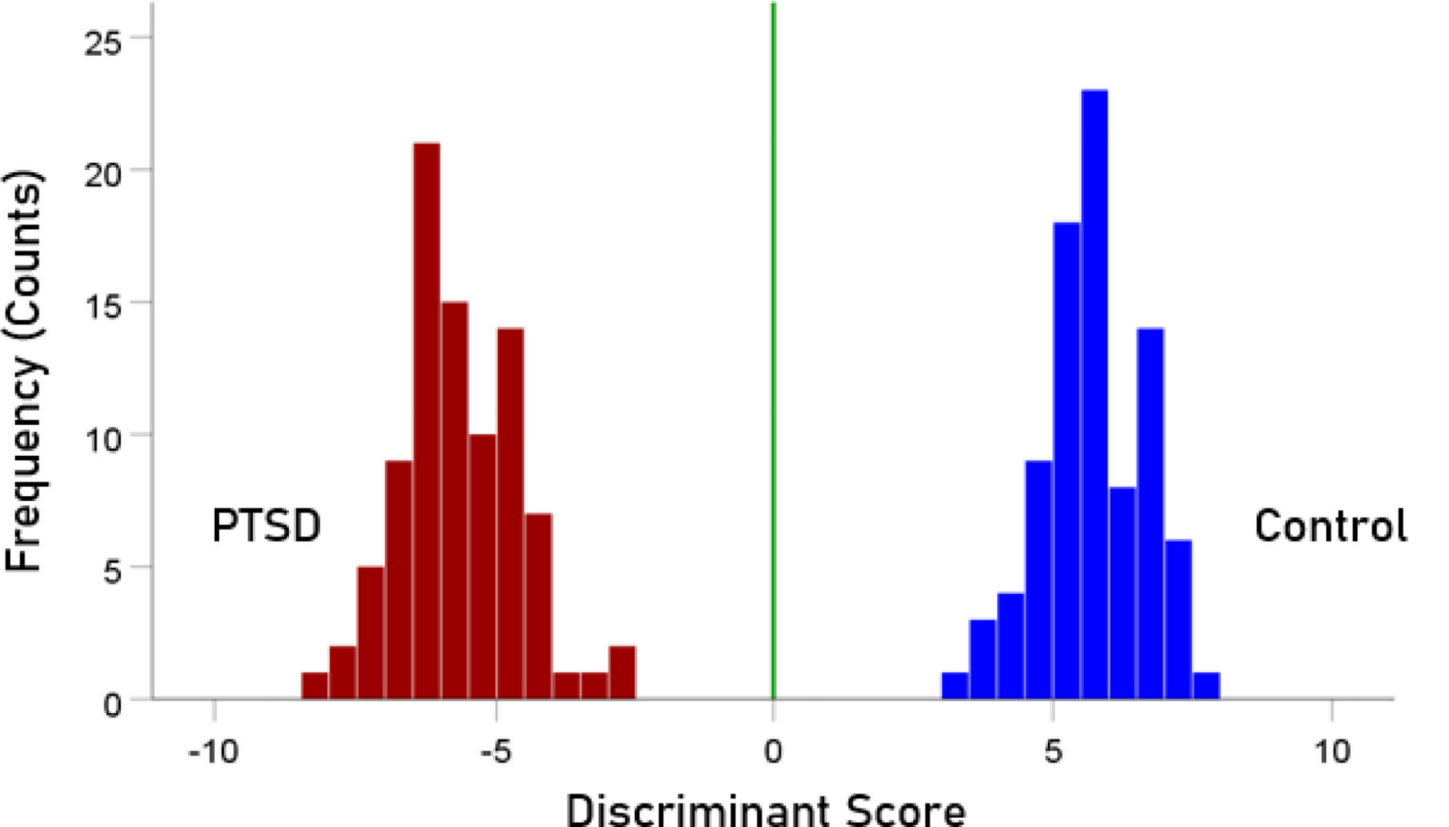
Frequency distribution of discriminant scores for the PTSD (N=88) and control (N=87) group.

**Figure 2.**
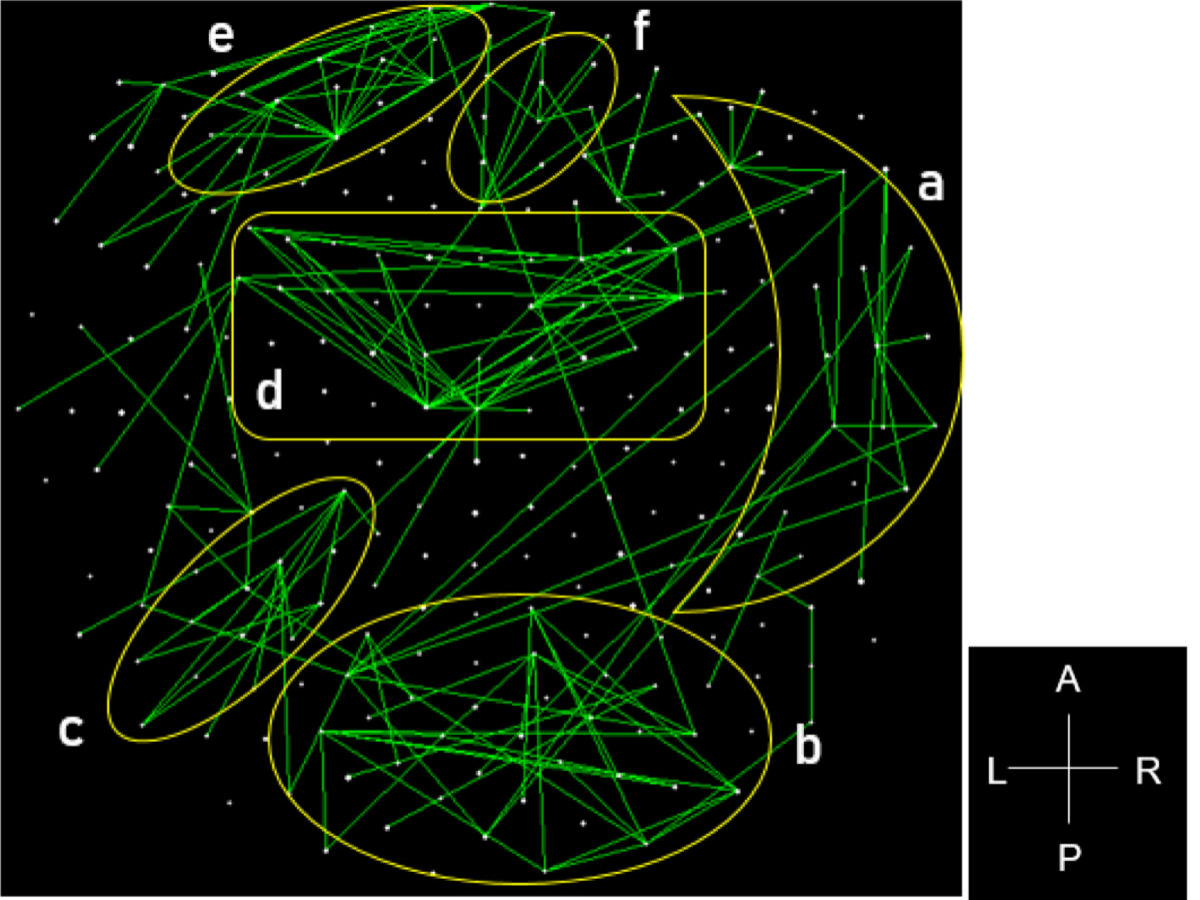
Functional connectivity networks from the 48 LDA feature-sensors are shown in a MEG sensor-space map. Connections with |SNI| greater than the 25^th^ percentile (> 0.0288) of the |SNI| distribution are plotted. Six network clusters are distinguished. A, anterior; P, posterior; L, left; R, right.

**Figure 3.**
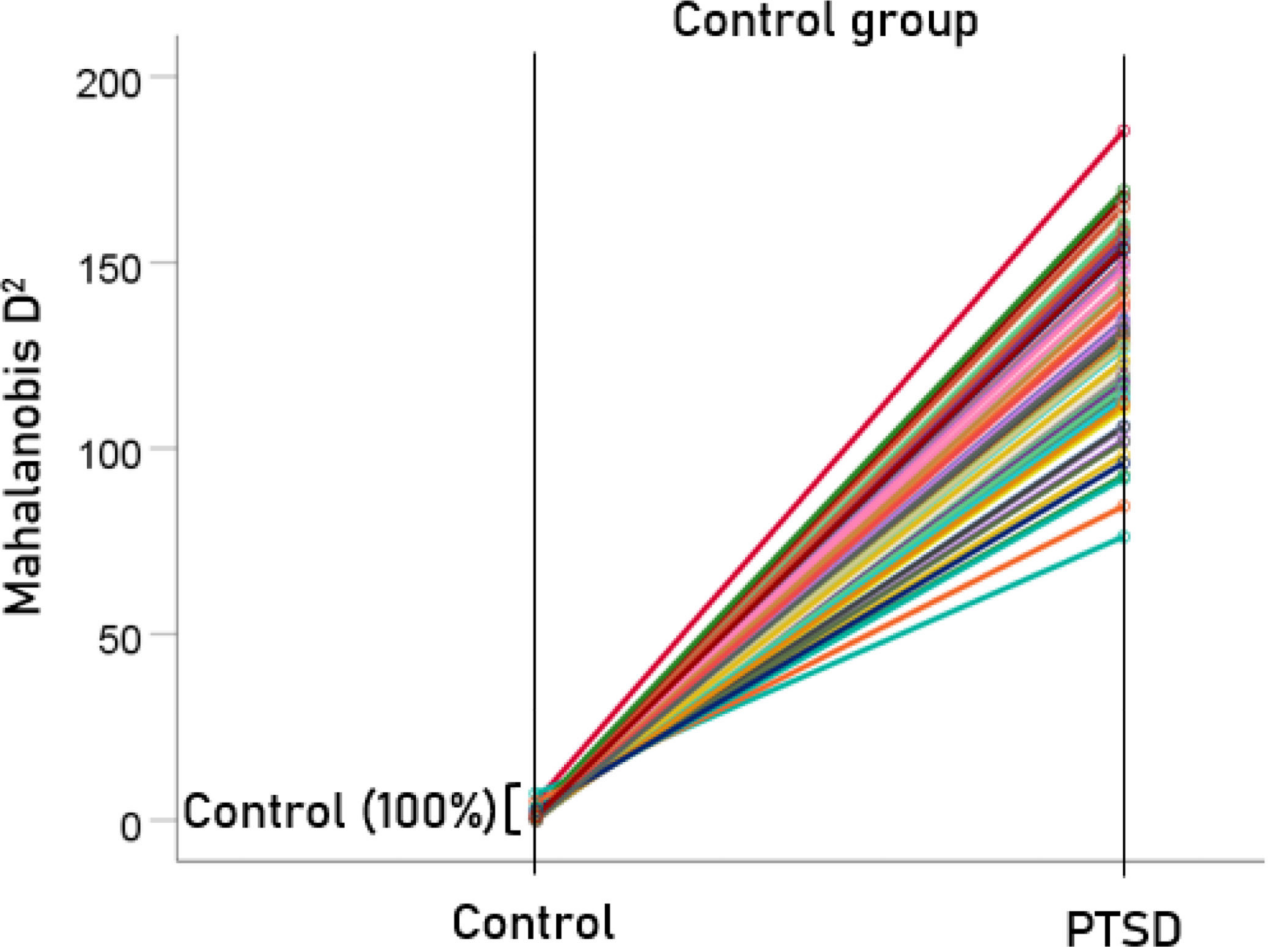
Mahalanobis ${\text{D}}^{2}$ values for the control group (N=87).

**Figure 4.**
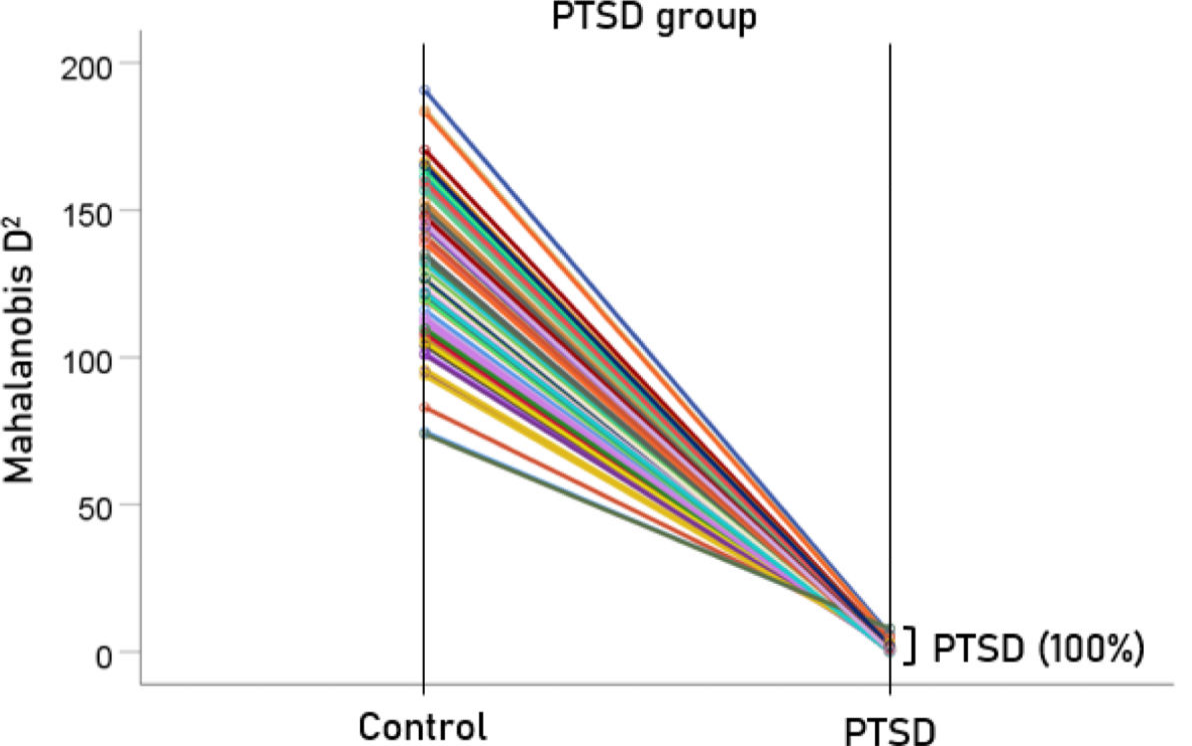
Mahalanobis ${\text{D}}^{2}$ values for the PTSD group (N=88).

The stepwise LDA yielded 100% correct classification of all 87 control and 88 PTSD brains with a probability of 1 for each brain, using 48/496 (9.68%) of the SNI predictors. A 100% correct classification was also obtained in a cross-validation leave-one-out test. The frequency distribution of the discriminant scores for control and PTSD brains are shown in Fig. 1. It can be seen that they were tightly clustered and did not overlap. Fig. 2 shows the 6 distinct functional connectivity networks identified by the 48 feature-sensors yielded by the LDA.. The ${\text{D}}^{2}$ Mahalanobis distances of the 257 control and PTSD cases are shown in Figs. 3 and 4, respectively. The tight cluster of control and PTSD values, respectively, attest to the high certainty of the classification outcome. Because women are known to be underrepresented in medical studies and can differ from males, we tested for gender differences in the ${\text{D}}^{2}$ Mahalanobis distances between women and men; there were no significant differences (Table 1).

### Classification of subthreshold, partially recovered, and fully recovered PTSD brains

**Figure 5.**
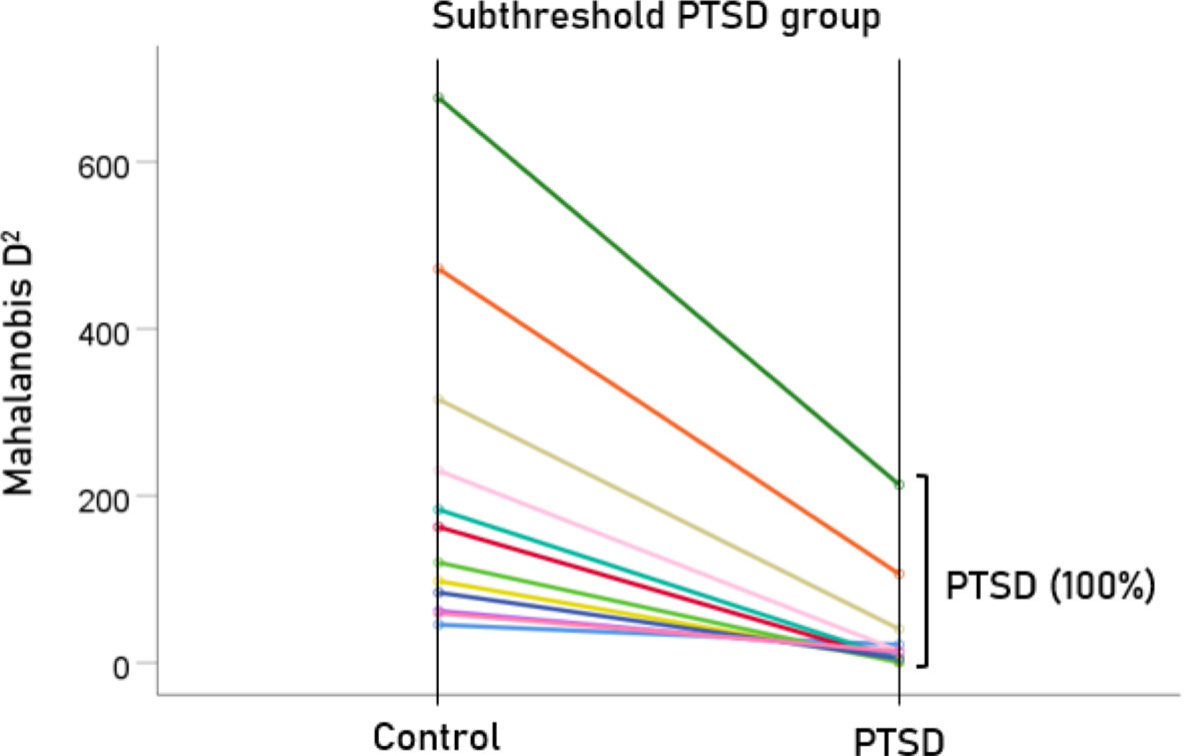
Mahalanobis ${\text{D}}^{2}$ values for the subthreshold PTSD group (N=13).

**Figure 6.**
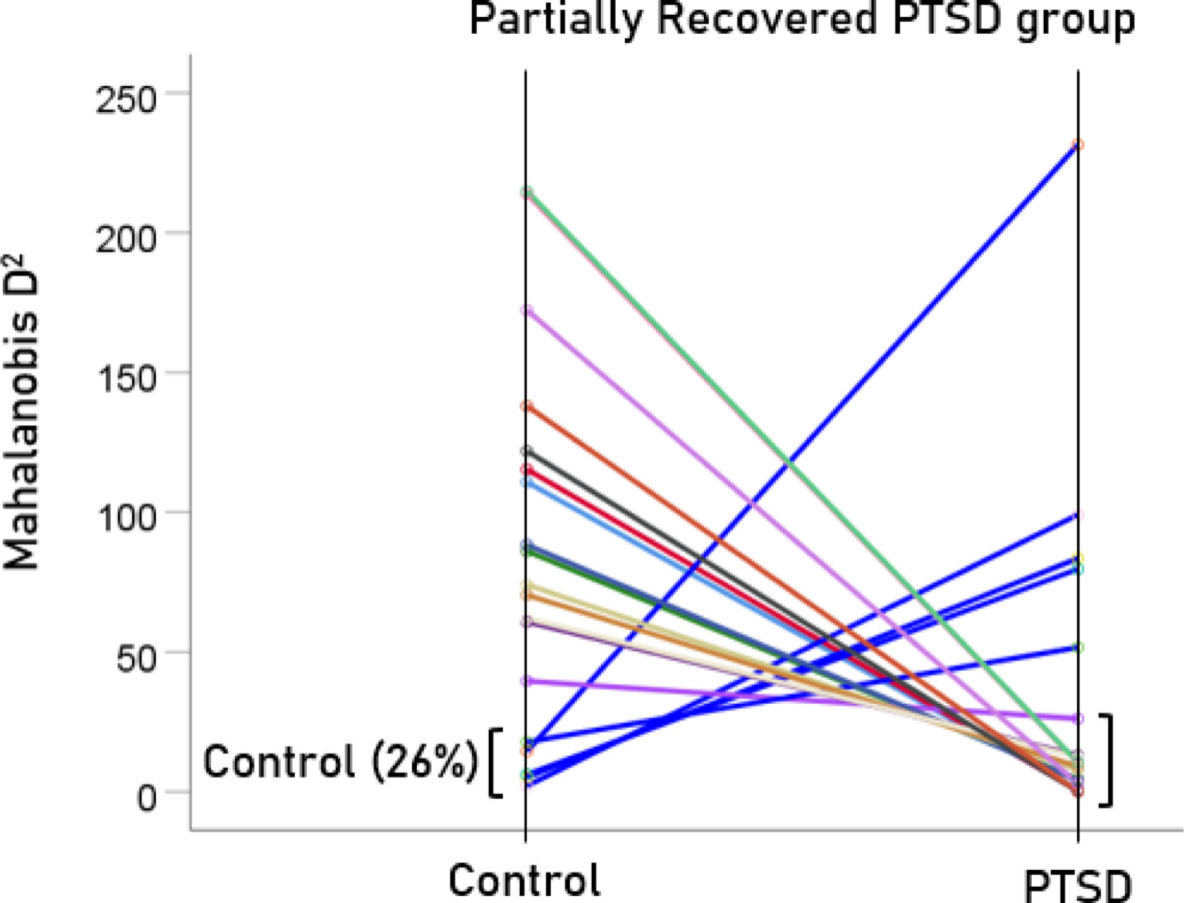
Mahalanobis ${\text{D}}^{2}$ values for the partially recovered PTSD group (N=19).

**Figure 7.**
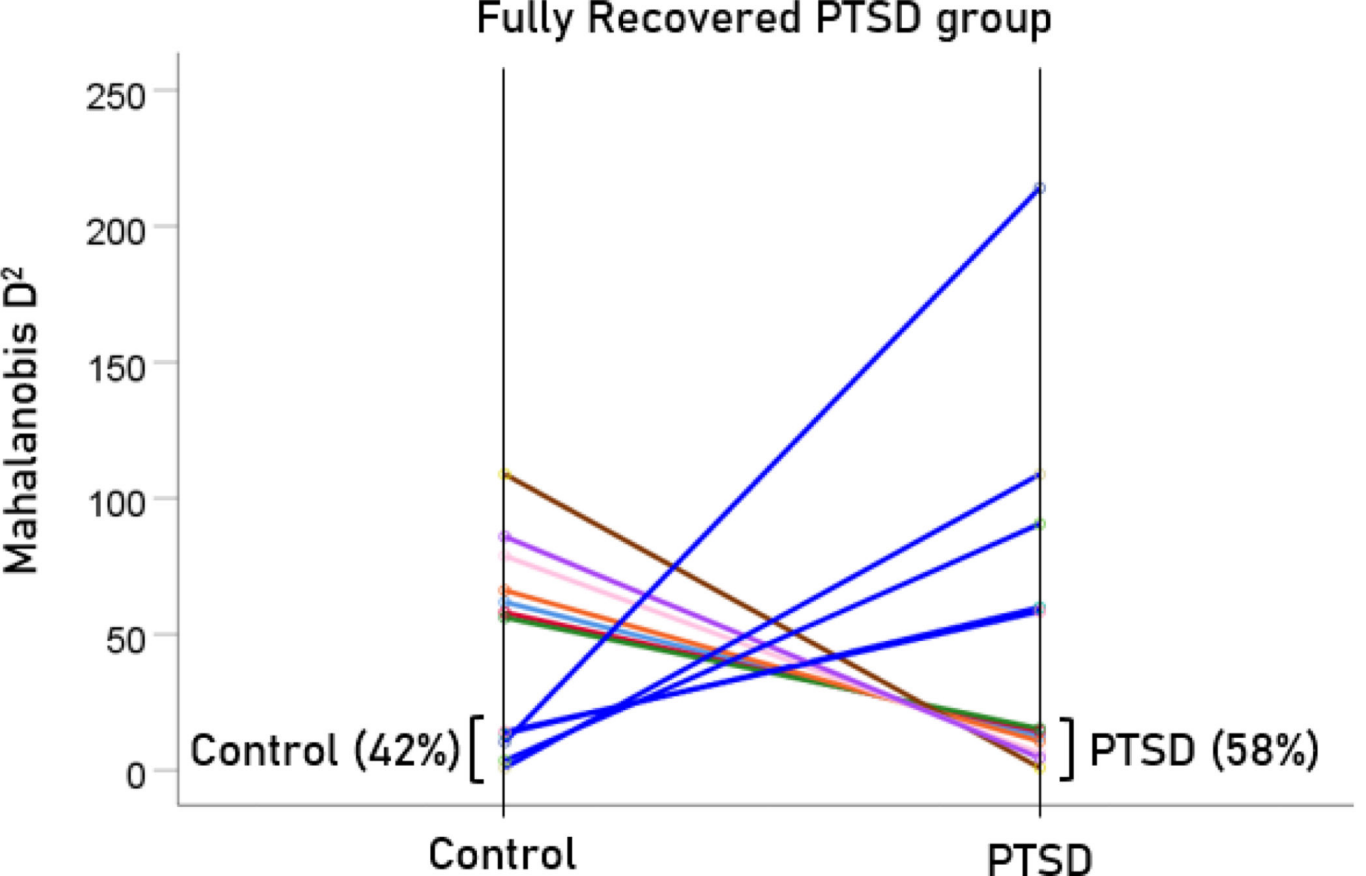
Mahalanobis ${\text{D}}^{2}$ values for the fully recovered PTSD group (N=12).

After having found that SNI can distinguish PTSD patients from controls with high certainty, we applied the same LDA model to the subthreshold, partially recovered, and fully recovered groups. All 13 subthreshold PTSD brains were assigned to the PTSD group (Fig. 5). All probabilities of classification were 1. Of the 19 partially recovered PTSD brains, 5 (26.3%) were classified as recovered PTSD brains, 5 (26.3%) were classified as control and 14 (73.7%) were classified as PTSD (Fig. 6). All probabilities of classification were 1. Of the 12 fully recovered PTSD brains, 5 (42%) were classified as control and 7 (58%) were classified as PTSD (Fig. 7). All probabilities of classification were 1.

### Normalized Mahalanobis D2 values

**Figure 8.**
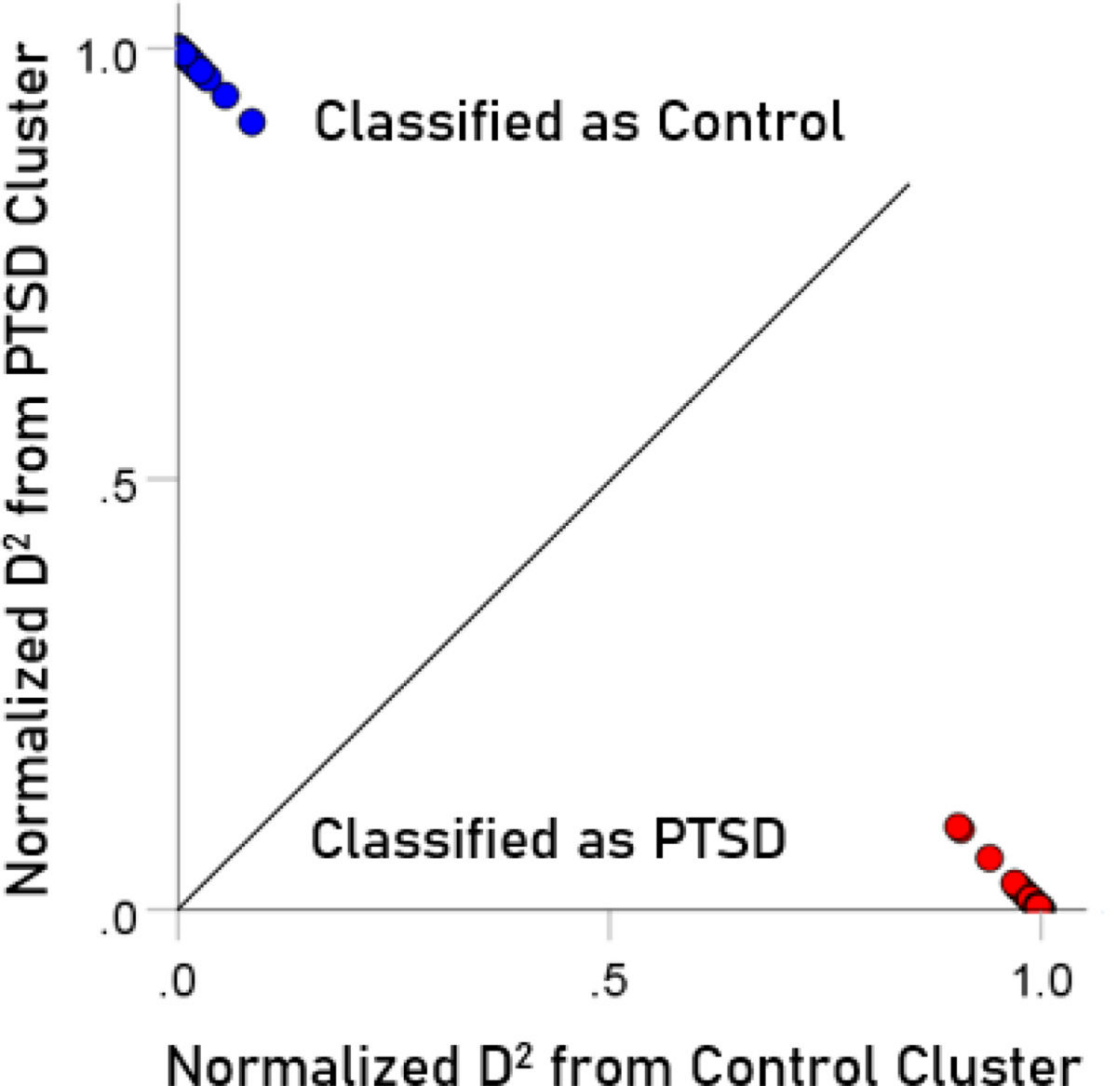
Normalized Mahalanobis ${\text{D}}^{2}$ values for the control and PTSD groups. (See text for details.)

**Figure 9.**
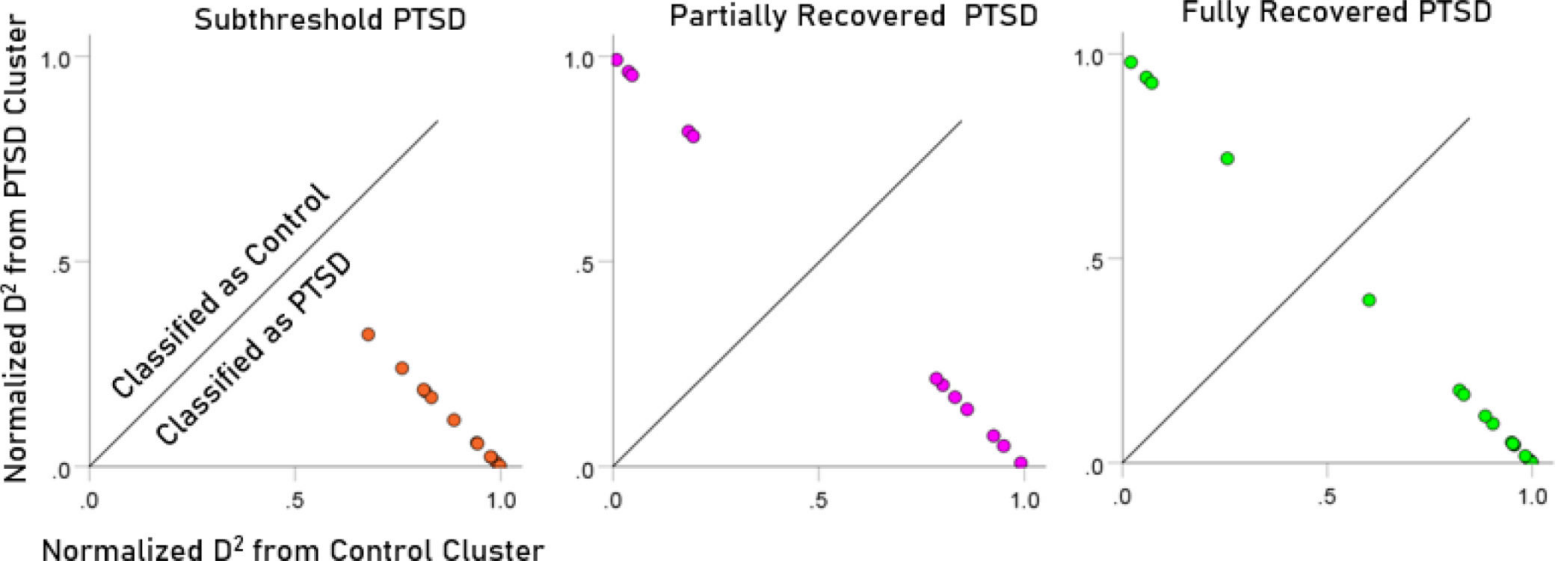
Normalized Mahalanobis ${\text{D}}^{2}$ values for the subthreshold, partially recovered, and fully recovered PTSD groups. (See text for details.)

The normalized Mahalanobis ${\text{D}}^{2}$ values for the control and PTSD groups are plotted color-coded in Fig. 8. It can be seen that they are clustered at the two extremes of the plot, attesting to the high certainty of classification. Fig. 9 (left panel) plots the normalized ${\text{D}}^{2}$ values for the subthreshold PTSD brains. It can be seen that they all lie within the PTSD area but are more spread that the PTSD values in Fig. 8, attesting to the less severe PTSD effect. Fig. 9 (middle panel) plots the normalized ${\text{D}}^{2}$ values for the partially recovered PTSD brains. It can be seen that they spread across both control and PTSD regions. A similar split classification outcome is seen in Fig. 9 for the fully recovered group.

### Association between PCL-5 score and D2 values

**Figure 10.**
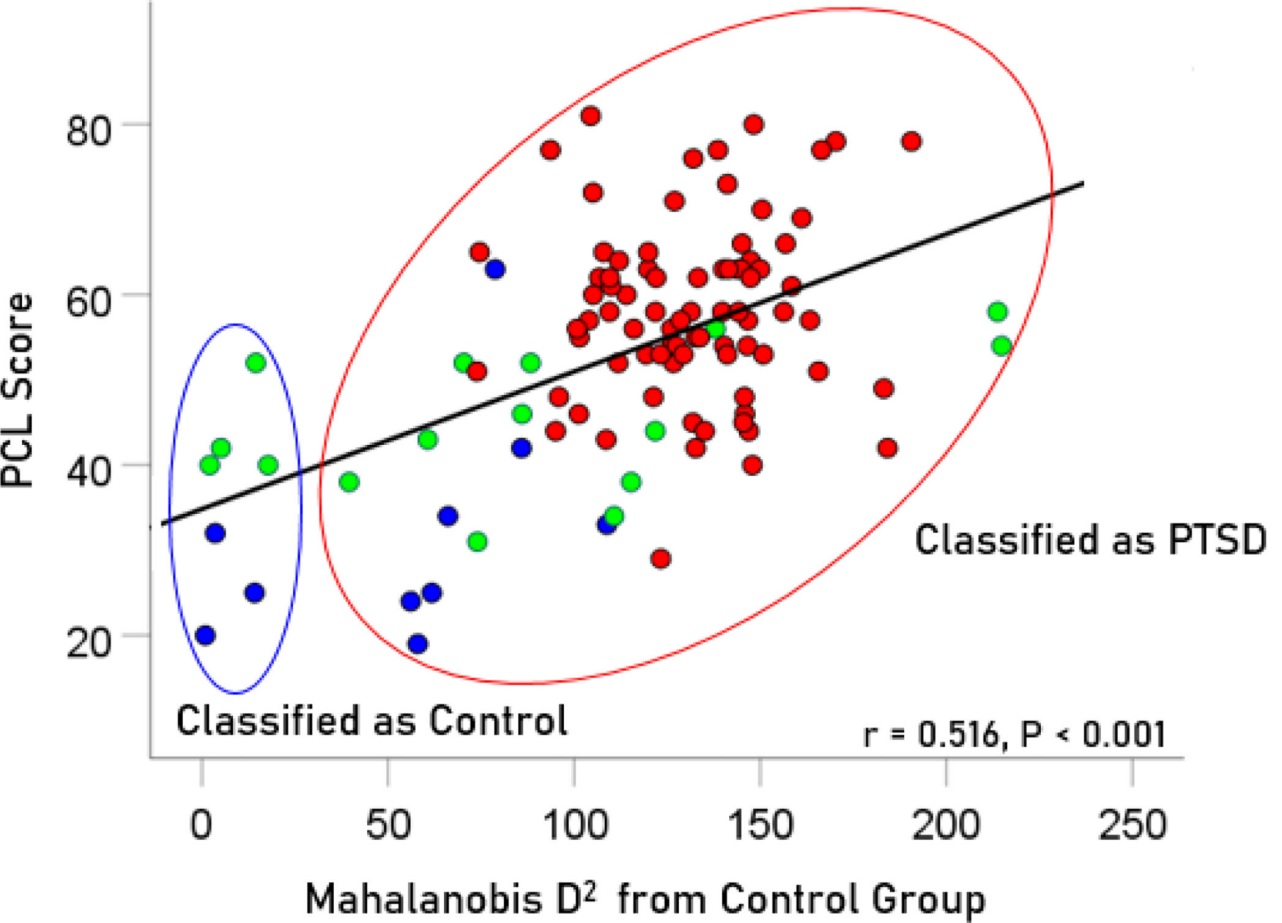
PCL scores for PTSD, partially and fully recovered PTSD are plotted against Mahalanobis ${\text{D}}^{2}$ distance from the control cluster; Pearson correlation r=0.516, P<0.001, N=106. Blue, fully recovered (n=10 PCL scores available of 12 total); green, partially recovered (n=16 PCL scores available of 19 total); red, PTSD (n = 80 PCL scores available of 88 total).

To evaluate the correspondence between the classification outcome and PTSD severity scores, the association between 106 PCL-5 scores and Mahalanobis ${\text{D}}^{2}$ values were evaluated for participants with PTSD (n=80 PCL scores available out of 88 total), partially recovered PTSD (n=16 PCL scores available out of 19 total), and fully recovered PTSD (n=10 PCL scores available out of 12 total). We found a highly significant positive correlation (r=0.516, P<.001, N=106) between PCL scores and the ${\text{D}}^{2}$ distance from the control cluster (Fig. 10). Finally, we compared the PCL scores between those classified as Control vs. those classified as PTSD and found that the former had highly significantly lower PCL scores than the latter (Table 2).

These findings further validate our approach and attests to the utility of the ${\text{D}}^{2}$ measure in assessing PTSD recovery.

## Discussion

In the present study we used MEG SNI to classify the functional brain status of US veterans diagnosed with subthreshold, partially recovered and fully recovered PTSD relative to a Control and PTSD group. The findings highlight brain functional similarities between subthreshold PTSD and PTSD and document variability in the functional brain status of PTSD-recovery trajectories. Overall, the findings add to the literature supporting the discriminatory power of MEG SNI and demonstrate the utility of SNI as a biomarker of PTSD-related status. The specific findings and their implications are discussed below, emphasizing the importance of objective indicators of PTSD.

Consistent with prior MEG-SNI classification studies^22–24^, the present findings indicate highly accurate classification of control and PTSD groups based on a small number of sensors (<10% of the total number of SNI predictors). Prior studies have demonstrated that SNI anomalies involving the right superior temporal gyrus and posterior-occipital regions distinguish PTSD from controls^25^. Here we documented that a relatively small number of sensors that distinguish veterans with PTSD from Controls also classified with 100% accuracy subthreshold PTSD as PTSD. That is, at least in terms of neural communication, brain functioning in individuals with subthreshold PTSD mimics that of individuals meeting full diagnostic criteria for PTSD. This finding provides objective evidence of brain dysfunction in subthreshold PTSD and highlights the need for additional clinical and research resources aimed at identifying subthreshold PTSD and reducing the distress and impairment associated with subthreshold PTSD symptoms.

While 100% of the subthreshold PTSD group was classified as PTSD, the partial and full recovery groups were not as clearly distinguished. Approximately 74% of the partial recovery group (i.e., those meeting lifetime criteria for PTSD and still exhibiting symptoms albeit not enough to meet current full PTSD criteria) were indeed classified as PTSD. That is, in terms of brain functional status, their brains were similar to those with full PTSD. The remaining 16%, though, were classified as Controls indicating absence of PTSD-related dysfunction. For the veterans defined as fully recovered based on diagnostic interviews, 42% were in fact classified as Control, yet more than half were classified as PTSD according to their patterns of brain function. Notably, those classified as Controls had significantly lower PCL scores than those classified as PTSD; however, the discrepancies between subjective symptom report and brain-based classification are noteworthy and merit additional discussion.

First, let us consider those classified as Control based on objective measures yet continue to report PTSD symptoms. One possibility is that the PTSD neural signature underlies specific PTSD symptoms. For example, previous MEG SNI studies of PTSD have demonstrated anomalies primarily involving the right superior temporal gyrus, an area previously associated with re-experiencing type phenomena^39^, distinguish PTSD from Controls^25^. Thus, it may be that individuals no longer have re-experiencing symptoms, a requisite for PTSD diagnosis, but continue to report other PTSD symptoms that may not be PTSD-specific (e.g., dysphoria or arousal symptoms). Another possible explanation for PTSD symptom reporting among those classified as Control according to brain function is symptom over-reporting. There are many reasons for symptom over-reporting among veterans including desire for care, compensation-seeking, and views about the military and/or government^19^. Conversely, those reporting full recovery yet brain measures classify them as PTSD may be under-reporting symptoms. Reasons for under-reporting include denial, stigma/shame associated with mental health concerns, views that mental health problems reflect weakness, and avoidance, among others^19^. An alternative explanation is that some individuals may develop a neural scar reflecting aberrant brain functioning resulting from traumatic experiences. In fact, one prior study demonstrated that veterans with PTSD in remission exhibited similar neural anomalies as those with PTSD, albeit attenuated^25^. In that case, it is possible that the neural system is primed such that subsequent exposure to potentially traumatic events results in re-emergence of PTSD-related symptoms. While we cannot say with certainly which of these or other possible explanations account for the discrepancies between objective and subjective reports documented in the present study, we do contend that the very existence of these discrepancies highlights the importance of using objective indicators.

The potential benefits and utility of PTSD biomarkers has been thoroughly described elsewhere^20^. These include usefulness across settings including clinical, forensic, and disability contexts, enhancing diagnostic specificity, counteracting over- and under-reporting, treatment planning, monitoring treatment response, and reducing stigma, among others. MEG SNI is well-suited for these applications (as are similar functional magnetic resonance imaging classification approaches based on cross-correlated neural networks^40^). That said, biomarker implementation must be balanced with consideration of potential costs that include invalidation of suffering in the absence of biomarkers, interpretation of the presence of a biomarker as a sign of permanent disability, and other iatrogenic effects^20^. It is our perspective that for PTSD the benefits of objective biomarkers outweigh the costs.

The present study advances the field by demonstrating the utility of applying the MEG SNI test across different PTSD trajectories. Although the findings have important clinical, research, and legal implications, they must be considered within the context of study limitations. First, although the findings were robust, the group sample sizes were relatively small and few women veterans participated in this study. Although the sample composition is reflective of the disproportionately male veteran population, the small number of women limits the generalizability of the present findings to the broader population of female veterans. Future studies with larger, more diverse samples are warranted to validate the current findings and evaluate potential moderating effects. Second, the present sample was selected to be free of other Axis I disorders; therefore, it is likely not representative of trauma-exposed U.S. veterans. Although we have demonstrated the validity of MEG SNI for classifying PTSD in those with and without comorbid conditions^23,24^, future studies including veterans with co-occurring mental health conditions are need for establishing the robustness of the MEG SNI PTSD biomarker. Finally, the current study was based on cross-sectional assessment that included retrospective reporting of symptoms. Longitudinal studies documenting posttraumatic changes in brain function and corresponding symptom reports are needed to further support MEG SNI’s use as an objective indicator of PTSD-related outcomes.

## Figures and Tables

**Table 1.** Comparison of ${\text{D}}^{2}$ Mahalanobis distances between men and women.

	${\text{D}}^{2}$ distances from the Control cluster (mean ± SEM)		P-value (t-test)
	Men	Women	
Control group (N = 87)	0.927 ± 0.141 (N=80)	0.311 ±0.187 (N=7)	P = 0.206 (NS)
PTSD group (N = 88)	129.083 ± 2.614 (N=74)	142.123 ± 7.553 (N=14)	P = 0.060 (NS)

**Table 2.** Comparison of PCL-5 scores between participants classified as Control vs.those classified as PTSD (Fig. 10).

PCL-5 scores (mean ± SEM)		P-value (t-test)
Classified as Control	Classified as PTSD	
35.86 ± 4.13 (N = 7)	55.04 ± 4.27 (N = 99)	P < 0.001

## Data Availability

Data available upon request.

## References

[1] American Psychiatric Association. Diagnostic and Statistical Manual of Mental Disorders (5th ed.). 2013.

[2] Elhai JD, North TC, Frueh BC. Health service use predictors among trauma survivors: a critical review. Psychol Serv. 2005; 2(1):3–19. doi:10.1037/1541-1559.2.1.3

[3] Kessler RC. Posttraumatic stress disorder: the burden to the individual and to society. J Clin Psychiatry. 2000; 61(suppl 5):4–12. 10761674

[4] Koenen KC, Stellman SD, Sommer JF Jr, Persisting posttraumatic stress disorder symptoms and their relationship to functioning in Vietnam veterans: a 14-year follow-up. J Trauma Stress. 2008; 21(1):49–57. doi:10.1002/jts.20304 18302174 PMC2654776

[5] Kubzansky LD, Koenen KC, Spiro A III, Prospective study of posttraumatic stress disorder symptoms and coronary heart disease in the Normative Aging Study. Arch Gen Psychiatry. 2007; 64(1):109–116. doi: 10.1001/archpsyc.64.1.109 17199060

[6] Kulka RA, Schlenger WE, Fairbank JA, Trauma and the Vietnam war generation: Report of findings from the National Vietnam Veterans Readjustment Study. Brunner/Mazel; 1990

[7] Kang HK, Natelson BH, Mahan CM, Post-traumatic stress disorder and chronic fatigue syndrome-like illness among Gulf War veterans: a population-based survey of 30,000 veterans. Am J Epidemiol. 2003; 157(2):141–8 doi:10.1093/aje/kwf187. 12522021

[8] Tanielian T, Jaycox L. Invisible wounds of war: Psychological and cognitive injuries, their consequences, and services to assist recovery. Rand Corporation; 2008.

[9] Wisco BE, Marx BP, Wolf EJ, Posttraumatic stress disorder in the US veteran population: results from the National Health and Resilience in Veterans Study. J Clin Psychiat. 2014; 75(12):1338–46. doi: 10.4088/JCP.14m09328. PMC9040390 25551234

[10] Jakupcak M, Conybeare D, Phelps L, McFall ME. Anger, hostility, and aggression among Iraq and Afghanistan war veterans reporting PTSD and subthreshold PTSD. J Trauma Stress. 2007; 20:945–954. doi: 10.1002/jts.202587 18157891

[11] El-Gabalawy R, Blaney C, Tsai J, Physical health conditions associated with full and subthreshold PTSD in US military veterans: Results from the National Health and Resilience in Veterans Study. J Affect Disorders. 2018; 227:849–53. doi: 10.1016/j.jad.2017.11.058 29689700 PMC6269149

[12] Mota NP, Tsai J, Sareen J, High burden of subthreshold DSM-5 post-traumatic stress disorder in US military veterans. World Psychiatry. 2016; 15(2):185. doi:10.1002/wps.20313 27265715 PMC4911785

[13] Bergman HE, Przeworski A, Feeny NC. Rates of subthreshold PTSD among US military veterans and service members: A literature review. Mil Psychol. 2017; 29(2):117–27. doi:10.1037/mil0000154 28630531 PMC5473625

[14] Bonanno GA. Loss, trauma, and human resilience: have we underestimated the human capacity to thrive after extremely aversive events? Am Psychol. 2004; 59(1):20–28. doi: 10.1037/0003-066X.59.1.20 14736317

[15] Bonanno GA, Mancini AD. Beyond resilience and PTSD: mapping the heterogeneity of responses to potential trauma. Psychol Trauma. 2012; 4(1):74–83. doi: 10.1037/a0017829

[16] Galatzer-Levy IR, Huang SH, Bonanno GA. Trajectories of resilience and dysfunction following potential trauma: A review and statistical evaluation. Clin Psychol Rev. 2018; 63:41–55. doi: 10.1016/j.cpr.2018.05.008 29902711

[17] Bonanno GA, Mancini AD, Horton JL, Millennium Cohort Study Team. Trajectories of trauma symptoms and resilience in deployed US military service members: Prospective cohort study. Brit J Psychiat. 2012; 200(4):317–23. doi:10.1192/bjp.bp.111.096552 22361018

[18] Donoho CJ, Bonanno GA, Porter B, A decade of war: prospective trajectories of posttraumatic stress disorder symptoms among deployed US military personnel and the influence of combat exposure. Am J Epidemiol. 2017; 186(12):1310–8. doi:1093/aje/kwx318 29036483 10.1093/aje/kwx318

[19] Guina J, Welton RS, Broderick PJ, DSM-5 criteria and its implications for diagnosing PTSD in military service members and veterans. Curr Psychiat Rep. 2016; 18(5):43. doi:10.1007/s11920-016-0686-1 26971499

[20] Lehrner A, Yehuda R. Biomarkers of PTSD: military applications and considerations. Eur J Psychotraumato. 2014; 5:23797. doi:10.3402/ejpt.v5.23797 PMC4138702 25206945

[21] Zoladz PR, Diamond DM. Current status on behavioral and biological markers of PTSD: a search for clarity in a conflicting literature. Neurosci Biobehav R. 2013; 37(5):860–95. Doi: 10.1016/j.neubiorev.2013.03.024 23567521

[22] Georgopoulos AP, Karageorgiou E, Leuthold AC, Synchronous neural interactions assessed by magnetoencephalography: a functional biomarker for brain disorders. J Neural Eng. 2007; 4(4):349. doi:10.1088/1741-2560/4/4/001 18057502

[23] Georgopoulos AP, Tan HM, Lewis SM, The synchronous neural interactions test as a functional neuromarker for post-traumatic stress disorder (PTSD): a robust classification method based on the bootstrap. J Neural Eng. 2010; 7(1):016011. doi: 10.1088/1741-2560/7/1/016011 20086271

[24] James LM, Belitskaya-Lévy I, Lu Y, Development and application of a diagnostic algorithm for posttraumatic stress disorder. Psychiat Res – Neuroim. 2015; 231(1):1–7. doi:10.1016/j.pscychresns.2014.11.007 25433425

[25] Engdahl B, Leuthold AC, Tan HM, Post-traumatic stress disorder: a right temporal lobe syndrome? J Neural Eng. 2010; 7(6):066005. 10.1088/1741-2560/7/6/066005 20980718

[26] Zhang J, Richardson JD, Dunkley BT. Classifying post-traumatic stress disorder using the magnetoencephalographic connectome and machine learning. Sci Rep. 2020; 10:5937. doi:10.1038/s41598-020-62713-5 32246035 PMC7125168

[27] DSM-IV-TR American Psychiatric Association. Diagnostic and Statistical Manual of Mental Disorders (4th ed., text rev.); 2000.

[28] Blake DD, Weathers FW, Nagy LM, The development of a clinician-administered PTSD scale. J Trauma Stress. 1995; 8(1):75–90. 7712061 10.1007/BF02105408

[29] First MB, Spitzer RL, Gibbon M, Structured Clinical Interview for DSM-IV-TR Axis I Disorders, Research Version, Non-patient Edition (SCID-I/NP). New York: Biometrics Research, New York State Psychiatric Institute; 2002.

[30] Adler AB, Wright KM, Bliese PD, A2 diagnostic criterion for combat-related posttraumatic stress disorder. J Trauma Stress. 2008; 21(3):301–8. doi:10.1002/jts.20336 18553417

[31] Weathers FW, Ruscio AM, Keane TM. Psychometric properties of nine scoring rules for the Clinician Administered Posttraumatic Stress Disorder Scale. Psychol Assess. 1999; 11:124–133 doi:10.1037/1040-3590.11.2.124

[32] Blevins CA, Weathers FW, Davis MT, The Posttraumatic Stress Disorder Checklist for DSM-5 (PCL-5): Development and initial psychometric evaluation. J Trauma Stress. 2015; 28: 489–498. 26606250 10.1002/jts.22059

[33] IBM Corp. IBM SPSS Statistics for Windows, Version 23.0. Armonk, NY: IBM Corp; 2015.

[34] MATLAB and Statistics Toolbox Release 2015b, The MathWorks, Inc., Natick, MA, United States; 2015.

[35] Mahan MY, Leuthold AC, Georgopoulos AP. Spatiotemporal brain network analysis of healthy humans based on magnetoencephalography and functional MRI in the resting state. BMC Neuroscience. 2015; 16(Suppl 1): 155.

[36] Box GEP, Jenkins GM. Time Series Analysis: Forecasting and Control. San Francisco, CA: Holden-Day; 1976.

[37] Fisher RA. Statistical Methods for Research Workers. 13th ed. Edinburgh, Scotland: Oliver & Boyd; 1958.

[38] Engdahl BE, James LM, Miller RD, A magnetoencephalographic (MEG) study of Gulf War Illness (GWI). EBioMedicine. 2016; 12:127–32. doi:10.1016/j.ebiom.2016.08.030 27592598 PMC5078573

[39] Penfield W, Perot P. The brain’s record of auditory and visual experience: a final summary and discussion. Brain. 1963; 86(4):595–696. 14090522 10.1093/brain/86.4.595

[40] Christova P, James LM, Engdahl BE, Diagnosis of posttraumatic stress disorder (PTSD) based on correlations of prewhitened fMRI data: outcomes and areas involved. Exp Brain Res. 2015; 233(9):2695–705. doi:10.1016/j.pscychresns.2014.11.007 26070898

